# Hémophilie: état des lieux dans un service de pédiatrie dans la région de l'oriental du Maroc

**DOI:** 10.11604/pamj.2014.18.126.4007

**Published:** 2014-06-10

**Authors:** Noufissa Benajiba, Yousra EL Boussaadni, Mohammed Aljabri, Yassamine Bentata, Rim Amrani, Maria Rkain

**Affiliations:** 1 Service de Pédiatrie, Hôpital Al Farabi, CHU Mohamed VI, Université Mohamed I, Oujda, Maroc; 2 Laboratoire de Physiologie Université Mohamed I, Oujda, Maroc

**Keywords:** hémophilie A et B, arthropathie, facteur antihémophilique, programme de santé, Hemophilia A and B, arthropathy, antihemophilic factor, health program

## Abstract

Pour les pays en voie de développement, l'hémophilie continue d’être une maladie de conséquence médicale et sociale désastreuse. Le but de ce travail est d'analyser le suivi d'une cohorte de patients hémophiles. Patients et méthodes: étude prospective étalée sur deux années et menée au centre référent d'hémophilie dans la région de l'orient du Maroc. Ont été inclus tous les patients présentant une hémophilie confirmée et âgé de moins de 18 ans. Résultats: sur 16 hémophiles, Quinze patients présentait une hémophilie A, l’âge moyen des patients était de 6,18 ans, la forme sévère, représentait 20,7%, la forme modérée: 33,3% et la forme mineure: 40%. Les circonstances de découverte étaient post circoncisionnelle chez 53,3% des patients, 20,7% post traumatique, 20% à l’âge de la marche; la durée d’évolution variait entre 2 mois et 10 ans. L'hémarthrose a été décrite au niveau des genoux, coudes et chevilles, avec une moyenne allant de 2 à 5 fois par an; l'arthropathie a été remarquée dans 33,3%. Le bilan immunologique a révélé des facteurs circulant inhibant chez deux patients. Le traitement était à base d'antalgiques, de plasma frais congelé. L'administration de facteurs VIII recombinés a été instaurée chez 40,6% des patients (plus de 90% des formes modérées et graves), grâce au programme national de prise en charge des hémophiles. Le décès était noté dans un seul cas lié à une hémorragie cérébrale. Conclusion: Nous insistons sur l'intérêt du programme national de prise en charge des hémophiles dernièrement instauré qui pourrait améliorer les conditions de vie de ces enfants.

## Introduction

L'hémophilie est une affection ubiquitaire avec une incidence de 1/10000 de naissances [1, 2]. De nombreux progrès ont été réalisés ces dernières années sur tous les aspects diagnostiques et thérapeutiques de la prise en charge des patients hémophiles [3]. Pour les 80% des hémophiles qui vivent dans les pays en voie de développement, l'hémophilie continue d’être une maladie de conséquence médicale et sociale désastreuse; et cela face à l'indisponibilité du traitement par les concentrés des facteurs anti hémophiliques du fait de leur coût élevé et l'absence de couverture sociale chez la plupart des patients. C'est le cas dans notre pays le Maroc ou on estimerait le nombre d'hémophiles à 3000 en l'absence pour le moment d'un registre national. L'effort des différentes associations des patients, des équipes médicales ont abouti à la création d'un programme national de prise en charge des hémophiles depuis la fin de l'an 2012. Nos rapportons dans ce travail, notre expérience dans la prise en charge de l'hémophilie au service de pédiatrie au sein du centre de référence régional de l'orient du Maroc. Notre objectif était d’évaluer la morbidité de cette affection dans nos conditions de suivi.

## Méthodes

### Patients

Notre étude a porté sur 16 patients hémophiles colligés au sein du centre de référence régional d'hémophilie au service de pédiatrie de l'orient du Maroc depuis début 2011 jusquà décembre 2013. Les Critères d'inclusion étaient: un âge 18 ans - un Taux du facteur antihémophilique effondré - et un suivi régulier au sein du service Nous avons noté à travers une fiche d'exploitation: les données épidémiologiques, cliniques (évalués sur le type, la sévérité de l'hémophilie, l’âge des patients, l’âge du diagnostic, l'existence d'autres membres hémophiles dans la famille), les données thérapeutiques ainsi que les données évolutives en particulier la survenue de complications principalement orthopédiques. Notre centre organise un suivi régulier des hémopathies chroniques entre autre pour les patients hémophiles par un système d'hôpital du jour. Ces patients étaient venus soit directement au centre, soit nous ont été adressés par d'autres structures de la région de l'oriental.

### Méthodes

Nous avons réalisé une étude prospective longitudinale. Une fois le diagnostic fait, les patients étaient éduqués sur la manière de prendre en charge à domicile les évènements hémorragiques peu graves. Le traitement à domicile des hémarthroses et des hématomes consistait à immobiliser le membre atteint, puis à appliquer de la glace et à effectuer une mobilisation précoce de l'articulation dès l'arrêt de la douleur. Les hémorragies extériorisées peu graves étaient traitées par une pression locale continue. Tous les accidents étaient notés sur un cahier que le médecin consultait au cours des rendez-vous trimestriels. Pour les hémarthroses qui résistaient au traitement à domicile, les patients devaient se rendre au service où ils recevaient ou non, selon la gravité de l'hémorragie, une perfusion de plasma ou de concentrés de facteurs selon la disponibilité. La décision d'amener le patient à l'hôpital était prise après l'avis du médecin traitant ou l'infirmier du centre de traitement joint par téléphone.

La morbidité a été évaluée sur la fréquence annuelle des hémarthroses (nombre total, nombre par patient, nombre par articulation), leur relation avec le degré de sévérité de la maladie, la fréquence annuelle des hématomes musculaires et des hémorragies extériorisées (nombre total et nombre par patient), le lieu de la prise en charge et la nécessité de perfusion de plasma frais congelé ou administration de concentrés de facteurs en cas de disponibilité.

Les conséquences médico-sociales ont été appréciées sur la prévalence des séquelles articulaires, celle de l'antigène de surface du virus de l'hépatite B (Ag HBs) et des anticorps contre le virus de l'immunodéficience humaine (VIH). Le dépistage de ces agents infectieux a été réalisé par la technique Elisa sur un rythme de 6 mois. En plus des données précédentes, le retentissement de la maladie a été apprécié aussi par le taux d'absentéisme et d’échec scolaire. Les résultats ont été analysés par le biais de l'excel.

## Résultats

### Données épidémiologiques et socio économiques

Il s'agit de 15hémophiles de type A répartis comme suit: 3 cas d'hémophile sévère (20%), 5 hémophiles modérés (33%) et 7 hémophiles mineurs (47%) et un cas d'hémophilie B. L’âge moyen était de 6 ans et 2mois +/- 3,7 ans avec un pic entre 5 et 10 ans. La durée d’évolution varie entre 2 mois et 10 ans selon les patients avec une durée moyenne de 3 ans et 3mois +/- 2,5 ans. L'histoire familiale de l'hémophilie est retrouvée dans 44% des cas, alors que dans le reste des cas, la maladie est dite de Novo. La notion de consanguinité a été notée dans 25% des cas, Le tiers de nos patients n'avait pas de couverture sociale, 20% disposaient d'une assurance maladie obligatoire (AMO) et dans 47% il s'agissait du Régime pour les malades démunis (RAMED).

### Circonstances de découverte

La maladie a été révélée dans 50% des cas par des hémorragies post circoncisionelles, dans 25% des cas par des épisodes hémorragiques survenus après un traumatisme minime et dans le reste des cas soit 25% par des écchymoses au niveau des genoux à l’âge de la marche. Le nombre d'accidents hémorragiques par an varie entre 1 et 10 épisodes ceci selon la sévérité de la maladie avec 3 épisodes comme chiffre moyen toute forme confondue, avec une prédominance des hémarthroses dans 60%, suivis par les hématomes des parties molles dans 40% des cas alors que les hémorragies extériorisées notamment les épistaxis et les gingivorragies ont été observées dans 20% des cas. L’âge du diagnostic dans notre série est de 2,65 ans + /-2,36

### Manifestations cliniques

#### Manifestations hémorragiques

Les hémarthroses: La fréquence moyenne des hémarthroses par patient a été de 10 par an dans la forme sévère, 5 dans la forme modérée et 2 dans la forme mineure. La localisation la plus observée dans notre étude, est le genou dans 70% des cas, suivie par les chevilles et les coudes dans 50% des cas, alors que les autres localisations comme l’épaule, les hanches ont été rarement observées. Les hématomes des parties molles étaient notés dans 50%, les hémorragies gingivales étaient notées dans 20%.

Les hémorragies extériorisées: les localisations principales étaient représentées par: les plaies cutanées, les gingivorragies, les hémorragies après chute dentaire, les épistaxis, l'hématurie.

L'hémorragie cérébro méningée: la localisation cérébrale a été notée dans un cas d'hémophilie A modérée, cela suite à une exposition au soleil.

#### Donnés évolutives

**Figure 1:**
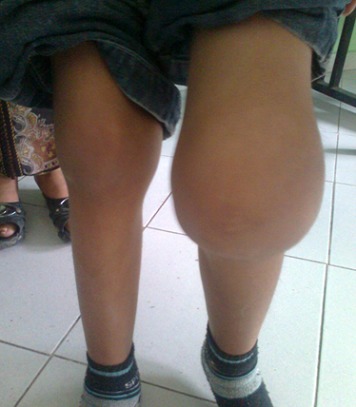
Hémarthrose du genou gauche chez un enfant hémophile de 6 ans

**Figure 2:**
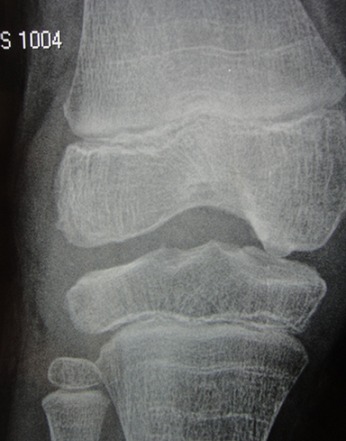
Arthropathie hémophilique du genou chez un enfant de 6 ans suivi pour hémophilie A majeure

L'arthropathie hémophilique: elle est observée chez 5 patients, soit 33%, elle siège essentiellement au niveau des genoux, avec déformation et boiterie et limitation du périmètre de marche chez un patient (Figure 1, Figure 2)

Complications infectieuses: la réalisation de sérologies virales a été la règle dans notre étude surtout chez les enfants polytransfusés, ainsi la recherche de l'antigène de surface du virus de l'hépatite B (Ag HBs), AC anti HVC des anticorps contre le virus de l'immunodéficience humaine (VIH) a été réalisé par la technique Elisa sur un rythme de 6mois, au cours de notre étude, le dépistage régulier n'a révélé aucun cas de conversion sérologique.

Complications immunologiques: deux hémophiles A sur les 15 patients suivis ont développé des anticorps anti facteur hémophilique VIII après la mise en route du programme.

Insertion sociale: sur les 16 patients suivis dans notre étude, l'absentéisme scolaire était noté chez tous les patients scolarisés, avec un cas d’échec scolaire dû aux épisodes rapprochés d'hémarthroses nécessitant des hospitalisations répétées.

Mortalité: un cas de décès a été noté dans notre série, suite à une hémorragie cérébro-méningée compliquée d'hydrocéphalie qui a bénéficié d'un acte chirurgical (drainage ventriculaire externe) sous couverture de facteurs selon les recommandations.

#### Donnés thérapeutiques

Prophylaxie: Un seul malade mutualiste bénéficie d'une prophylaxie primaire par le facteur recombinant à raison de 2 fois/semaine.

Traitement des accidents hémorragiques: Le recours à l'hospitalisation devant des épisodes hémorragiques était la règle surtout chez les patients ayant une forme majeure de la maladie. La prise en charge consistait généralement à la perfusion du plasma frais congelé vu le coût élevé des concentrés de facteurs VIII. Actuellement, grâce à la mise en route du programme national de prise en charge de l'hémophilie depuis 1 année, 7 de nos malades démunis sous régime du RAMED bénéficient dernièrement de l'administration des facteurs antihémophiliques VIII type recombinant pour les hémophiles A; soit plus que 90% des formes graves et modérées. Traitement des complications articulaires: réeducation fonctionnelle dans tous les cas, une synoviorthése: infiltration de corticoides dans trois cas (deux formes modérées et une forme grave) avec amélioration sauf dans un cas qui avait développé des inhibiteurs positifs.

## Discussion

L'hémophilie A est la plus fréquente, ce qui concorde avec les différentes études [1]. La consanguinité a été retrouvée dans 25% des cas: pays de forte consanguinité. Concernant les circonstances de découverte, l'hémorragie post circoncisionnelle a été le mode de révélation dans 50% des cas dans lesquels le bilan d'hémostase n'a pas été realisé ce qui n'est pas habituellement retrouvé, à noter que les patients habitant en milieu rural ont des difficultés à accéder aux soins et préfèrent consulter chez des guérisseurs traditionnels notamment pour une éventuelle circoncision. Les autres circonstances de découverte sont classiquement retrouvées tel que les hémorragies extériorisées ou les écchymoses à l’âge de la marche [4–7]. La localisation des hémarthroses a été respectivement plus fréquente au niveau du genou, du coude et de la cheville et ce conformément à d'autres études [5, 6]. La susceptibilité de ces articulations aux hémarthroses s'explique par le fait qu'elles n'ont qu'un seul plan de mobilité et que toute sollicitation en dehors de ce plan peut entrainer une élongation capsulo-synoviale, source d'hémorragie [4, 5, 7].

Concernant le volet thérapeutique, la décision de perfuser était toujours prise par un pédiatre et se fondait sur l'existence ou non de signes d'inflammation en cas de saignements non extériorisés, sur un saignement extériorisé persistant ou sur la gravité d'un traumatisme (exemple: traumatisme crânien). En l'absence de ces signes, le patient recevait un traitement antalgique. Il existe actuellement sur le marché marocain différents produits pour la prise en charge de l'hémophilie A: dérivé sanguin et recombinant par génie génétique tous remboursables par la sécurité sociale, mais le traitement a été à base de plasma frais congelé dans la plupart des cas vu que la quasi-totalité de nos malades sont indigents (20% ont une couverture sociale). Grace aux pressions des associations, des malades et des volontés politiciennes un programme de prise en charge des hémophiles a été instauré dernièrement depuis 1 année avec mise à la disponibilité des hôpitaux publics de facteur VIII recombinant gratuitement pour le traitement curatif des patients indigents qui bénéficient du régime RAMED. La prophylaxie primaire n'est pas concernée par ce programme ce qui fait que le problème des circoncisions est toujours soulevé.

Les conséquences médico-sociales sont dominées par les séquelles articulaires ce qui a déjà été noté dans des études antérieures [7] leur prise en charge est lourde et se base entre autres sur la réeducation fonctionelle, la synoviorthése, la synoviectomie [8–10]. Dans les pays développés le traitement prophylactique a réduit nettement la fréquence des hémarthroses et par conséquent de l'arthropathie hémophilique [10–12]. Dans les pays en voie de développement ou la prophylaxie ne peut être assurée vu le cout élevé des facteurs elles restent la complication fonctionnelle la plus redoutable source d'handicap majeur [10]. Dans notre étude elles étaient observées chez 5 de nos enfants soit 33,3% qui ont bénéficié tous de rééducation fonctionnelle, de synoviorthéses chez 2 de nos patients. Les complications infectieuses n'ont pas été retrouvées, aucune infection virale n'a été démontré chez nous en pédiatrie à savoir que tous nos enfants sont vaccinés contre l'hépatite B donc la contamination par des agents infectieux ne semble pas une préoccupation dans notre pays. Par contre les complications immunologiques sont bien la: depuis l'application du programme depuis deux ans déjà deux de nos patients sur 15 soit 13,3% ont développé un AC inhibiteur positif, et nous étions obligés de remettre les patients sous Plasma frais congelé à défaut de moyens; ce qui a poussé les médecins traitants à soulever ce problème avec le ministère de la santé pour discuter du choix du produit utilisé et dernièrement le produit dérivé sanguin a été instauré dans le programme [13]. Un cas de diabète a été retrouvé chez un patient porteur d'hémophilie modérée qui prenait la corticothérapie orale à titre antalgique pour des hémarthroses.

L'absentéisme scolaire est retrouvé chez la majorité des enfants surtout dans les formes sévères et l′échec scolaire dans un cas, ce qui rend compte des difficultés d'insertion sociale des hémophiles qui doivent bénéficier d'un soutien de la société. Autre difficulté est représentée par l′abstention de circoncision cette situation est très mal vécue par le patient et par ses parents chez qui cette pratique est considérée comme un acte d'une grande valeur religieuse et de reconnaissance sociale pour le jeune garçon dans un pays musulman.

Le décès a été noté chez un seul patient hémophilique A modéré âgé de 8 ans qui a présenté une hémorragie cérébrale suite à une exposition au soleil et en absence de toute prophylaxie: le patient a été opéré sous couverture du facteur avec des bilans de contrôle arrivant à un taux de facteur VIII à 80% mais le patient a succombé à J10 post opératoire par des complications post opératoires ce qui tire encore l'alarme sur la gravité de la maladie qui peut engager le pronostic vital dans les pays ou un traitement prophylactique ne peut être assurée que pour une minorité.

## Conclusion

L'hémophilie reste un problème de santé dans les pays en voie de développement, d'un coté par la gravité de certains tableaux hémorragiques qui peuvent entrainer le décès ou par le biais des complications articulaires entravant le pronostic fonctionnel. Et d'un autre coté par le cout élevé des facteurs. L'instauration récente d'un programme de prise en charge des hémophiles avec mise en disponibilité gratuite des facteurs et création des centres de référence équipés en personnel: kinésithérapeute, infirmière et médecin référent a fortement soulagé le personnel soignant ainsi que les familles des patients.
